# Flexible In-Sensor Computing Strain Sensor for Lower-Limb Gait Recognition

**DOI:** 10.3390/mi17060710

**Published:** 2026-06-10

**Authors:** Jiayu Ma, Yuyu Feng, Ye Tian, Hao Guo, Zongmin Ma

**Affiliations:** School of Semiconductor and Physics, North University of China, Taiyuan 030051, China; mjiayu2022@163.com (J.M.); fyuyu5235@gmail.com (Y.F.); guohao@nuc.edu.cn (H.G.)

**Keywords:** flexible in-sensor-computing strain sensor, strain sensor, lines-of-non-extension (LoNE), gait recognition, analog-domain classification

## Abstract

Flexible strain sensors have attracted considerable attention in gait recognition owing to their ability to adhere directly to the skin near joints and transduce local deformation. In existing work, however, sensor placement and orientation are largely determined by anatomical experience, while multi-channel classification still relies on back-end digital processors, whose power consumption and latency constrain system practicality in wearable scenarios. This paper presents an integrated design path that proceeds from skin-mechanics theory through sensor-layout optimization to analog-domain front-end inference. On the layout side, the lines-of-non-extension (LoNE) theory is employed to convert the selection of sensor attachment angles from empirical judgment into a calculable mechanics problem; guided by the spatial course of LoNE in the ankle and knee regions, the positions and angles of the nine sensors are determined individually—channels perpendicular to the LoNE capture maximum strain, channels offset by 45 degrees supplement non-sagittal-plane information, and a channel aligned along the LoNE provides a near-zero-strain reference. On the circuit side, the mathematical equivalence between the weighted summation of a linear classifier and Kirchhoff’s current law (KCL) nodal current superposition is exploited to map the classification operation onto current aggregation in an analog circuit, yielding an in-sensor computing (ISC) front end in which the nine-channel weighted summation is completed in a single analog step. The sensors are fabricated by screen-printing a liquid-metal–polymer composite conductive ink onto a TPU film substrate, with a gauge factor RSD of 6.8% and a tensile linearity $R^{2}>0.99$. Using walking, running, and stair descent as verification targets, the analog classifier reaches 99% accuracy at the circuit-level functional-verification stage. On real multi-subject data, it achieves 87.0%±8.4% accuracy under intra-subject cross-session validation, with an analog-domain inference response faster than 100μs. This design path is not bound to a specific joint or sensor material; when the layout methodology is extended to additional joint regions and the circuit architecture incorporates multiple outputs to cover more classification categories, the same workflow remains applicable, offering a promising low-power, lightweight technical solution for wearable motion monitoring.

## 1. Introduction

Gait monitoring technology plays an increasingly important role in rehabilitation assessment, sports-injury prevention, and daily health management [1,2,3]. A variety of lower-limb motion monitoring schemes have been developed for clinical and research use, including inertial measurement units (IMUs) [4], optical marker-based motion capture systems, and surface electromyography (sEMG) acquisition [5]. IMUs are conveniently worn yet sense whole-limb acceleration rather than local joint deformation; optical motion capture offers high spatial resolution but is confined to a fixed laboratory; sEMG is sensitive to inter-subject muscular variability and susceptible to electromagnetic interference. By contrast, flexible strain sensors adhere directly to the skin near joints and transduce local deformation into resistance changes, striking a favorable balance between signal specificity and wearable portability [6]. If the attachment position and angle of such sensors could be further guided by quantitative skin-mechanics evidence, and if the multi-channel classification could be moved from a back-end PC to the sensor front end, the practicality of these sensors in multi-joint real-time recognition scenarios would be substantially enhanced.

Resistive strain sensors based on various conductive materials—including carbon nanotubes (CNTs), silver nanoparticles (AgNPs), and liquid-metal (LM) composites—have been widely explored for wearable motion monitoring owing to their simple readout circuitry and high stretchability. However, each sensing mechanism carries distinct trade-offs in hysteresis and long-term stability. CNT- and AgNP-based resistive sensors are known to suffer from considerable hysteresis, baseline drift, and poor long-term stability, and nanoparticle-based sensors in particular are prone to electrical disconnection from inter-particle separation under strain, often requiring algorithmic compensation to recover accurate values [7,8]. LM-particle-based resistive sensors offer improved conductivity retention at large deformations, yet baseline drift after strains exceeding 100% has also been reported [9,10], indicating that hysteresis remains a non-trivial concern even for this material class. By contrast, capacitive sensors with interdigitated-electrode (IDE) configurations generally exhibit lower hysteresis and superior long-term stability, and may be preferable for applications demanding high deformation fidelity [9]. For the present application, skin deformation at the ankle and knee during walking, running, and stair descent remains within approximately 10–60% strain—a regime in which the LM resistive sensors used here exhibit low hysteresis and stable cyclic response. The ISC classification architecture further mitigates residual sensor-to-sensor variability through offline weight training, providing additional robustness against sensor non-idealities.

At the layout level, the mechanical behavior of skin is markedly anisotropic: in the vicinity of a single joint, tensile and compressive strains in different locations and directions can differ several-fold, so the attachment site and orientation of a sensor directly determine the amplitude and selectivity of the acquired signal. Nevertheless, the vast majority of existing studies select attachment points on the basis of anatomical experience alone, with sensor orientation rarely discussed [6]. In fact, skin mechanics has long possessed a directional quantification framework—the lines of non-extension (LoNE). In 1964, Iberall first pointed out that the human skin surface harbors a set of directional lines along which length remains unchanged during joint motion [11]. Subsequently, Bethke quantified the complete strain field of the knee region via three-dimensional laser scanning [12]; Wessendorf and Newman tracked the evolution of the lateral-knee LoNE direction with flexion angle using an infrared motion-capture system and found that its inclination relative to the limb axis remained stable between 60 and 85 degrees [13]; Marreiros carried out systematic measurements on the ankle region, identifying seven groups of LoNE [14]; Choi and Hong reproduced the spatial distribution of knee LoNE on six subjects [15]. All of these studies, however, applied LoNE exclusively to the structural design of space suits [11,12], prosthetic sockets [16], or functional sportswear [15]—the guiding principle being to lay inextensible fibers along the LoNE direction so as to avoid constraining body motion. Reversing this directional information—using it to guide the angle at which sensors should be attached for optimal signal acquisition—could provide a new approach and framework for motion detection.

At the signal-processing level, existing flexible-sensor systems generally rely on a serial architecture of front-end acquisition, Bluetooth relay, and PC-side classification [17,18]. The wireless transmission of multi-channel raw waveforms together with digital-domain classification imposes combined power and latency burdens that become increasingly difficult to accommodate in wearable scenarios where lightweight design and real-time response are paramount. The in-sensor computing (ISC) architecture offers an alternative path: the weighted summation required for classification is performed directly in the analog domain at the sensor front end, bypassing the data-movement bottleneck [19,20]. Mennel et al. achieved nanosecond-scale image classification on a MoS_2_ phototransistor array [21]; Gao et al. applied a similar concept to a diamond nitrogen-vacancy (NV) center quantum sensing system, attaining a magnetic-field identification response time of approximately 137 ns [22,23]; Liu et al. exploited the reservoir dynamics of electrolyte-gated transistors for near-sensor gait-pathology recognition [24]. The ISC architecture has been successfully applied in optoelectronic and quantum sensing, yet a scheme combining analog-domain classification with skin-mounted flexible strain sensors remains to be explored.

Against this background, the present work introduces LoNE theory into the layout design of flexible strain sensors. Drawing on experimentally measured LoNE directions in the ankle and knee regions, nine sensors are positioned and oriented so that core channels perpendicular to the LoNE capture maximum strain, exploratory channels offset by 45 degrees extract non-sagittal-plane information, and a coupling channel aligned with the LoNE doubles as a zero-strain reference. On this layout foundation, an analog-domain ISC classification front end is constructed, in which a five-stage adjustable-gain circuit maps the nine-channel weighted summation onto KCL current superposition, completing the classification operation in a single analog step. Channel-ablation experiments are further conducted to quantify the contribution of each sensor tier to classification performance, and a multi-dimensional comparison is made with several reported PC-based, MCU-based, and near-sensor computing schemes. On three daily gaits—walking, running, and descending stairs—the system achieves a classification accuracy of 99% with microsecond-level inference latency.

## 2. Principles and Design of the Classification and Recognition System

**Figure 1:**
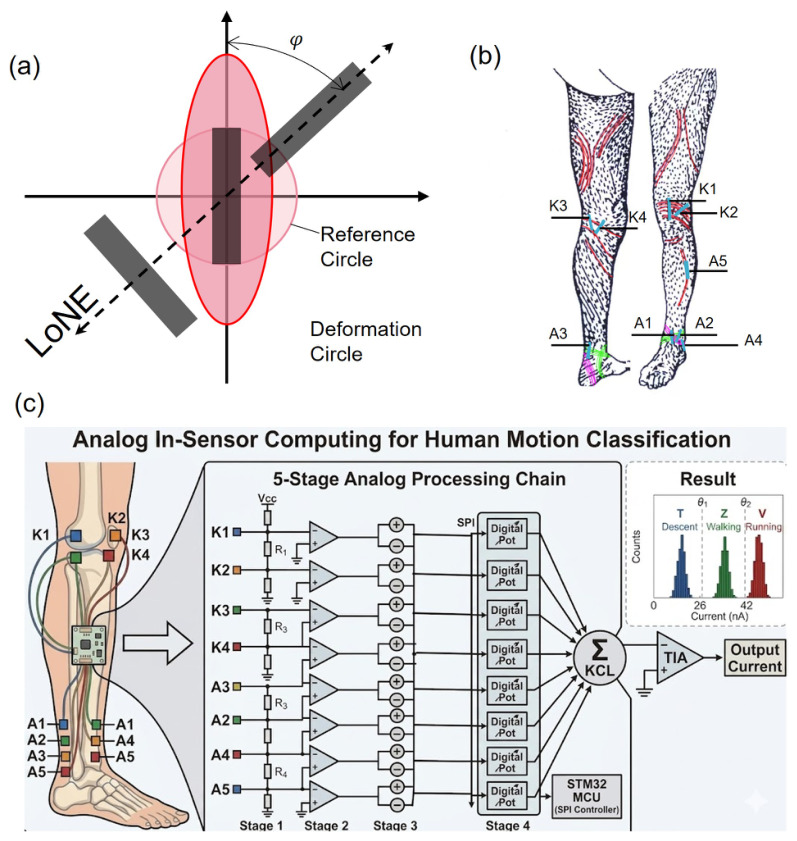
System design overview. (**a**) Schematic of the LoNE principle: Mohr-circle representation of the two-dimensional skin strain state; the intersections of the reference and deformation circles define one LoNE direction, with φ denoting the angle between the LoNE and the major principal-strain axis. (**b**) LoNE-guided placement of nine surface sensors on the lower limb, shown in anterior and posterior views. Red lines denote the spatial distribution of LoNE trajectories in the knee region; green and pink lines indicate the distribution of LoNE trajectories around the ankle complex. Sensor locations are highlighted by blue patches, with K1–K4 positioned about the knee, A1–A4 about the ankle, and A5 located along the mid-shank. (**c**) Five-stage analog-domain signal-processing architecture of the ISC front end: Stage 1 resistive-divider detection, Stage 2 instrumentation amplification, Stage 3 polarity encoding via non-inverting/inverting followers, Stage 4 digital-potentiometer weight scaling, and KCL current summation through a TIA to produce the classification output current.

The theoretical starting point of the layout design is the lines of non-extension (LoNE) in the skin strain field, defined as directions along which normal strain is exactly zero. As illustrated in Figure 1a, the two-dimensional strain state induced by joint motion can be described by a Mohr circle: the undeformed state corresponds to the reference circle and the deformed state to the deformation circle. When the maximum principal strain $\varepsilon_{I}$ > 0 (tension) and the minimum principal strain $\varepsilon_{II}$ < 0 (compression), the two circles intersect at two points whose directions are the LoNE—along these directions, the skin length remains unchanged before and after deformation. The angle φ between the LoNE and the major principal-strain axis is given by Equation (1) [14]:

(1) $$\tan^{2}\varphi =\frac{\varepsilon_{I}\left(2+\varepsilon_{II}\right)}{-\varepsilon_{II}\left(2+\varepsilon_{I}\right)}$$

Equation (1) establishes a deterministic mapping between the LoNE direction and the principal-strain magnitudes. On this basis, the attachment angle is selected individually for each of the nine sensor channels: channels mounted perpendicular to the LoNE have their sensitive axis aligned with the maximum-strain direction, yielding the largest resistance-change amplitude during joint flexion–extension; channels mounted along the LoNE theoretically produce no response to the motion and thus serve as zero-strain references and checkpoints for layout correctness; channels offset by 45 degrees fall between these two extremes, exploiting projection-component differences to capture non-sagittal-plane motion information missed by the perpendicular channels. The three angular tiers work in concert so that the array covers as many degrees of freedom as possible within a limited channel count.

Published skin strain-field data for the ankle [14] and knee [12,13,15] regions yield the spatial distribution of LoNE in each joint zone. In the ankle region, peak longitudinal tension during plantar flexion reaches 0.378 in the anterior zone while peak longitudinal compression reaches −0.463 over the Achilles tendon, with strictly opposite signs; Marreiros identified seven LoNE groups, of which L4, L5, and L6 follow a spiraling upward course in the distal shank [14]. In the knee region, the lateral LoNE direction subtends an angle of approximately 60 to 85 degrees with the longitudinal axis and remains highly stable throughout the flexion-extension cycle [13].

Three exploitable features emerge from the LoNE distribution in the ankle and knee regions: the principal-strain signs in the anterior ankle and Achilles zones are strictly opposite, and quantitative LoNE data are available in the literature for both; the lateral-knee LoNE direction holds steady at 60–85 degrees (standard deviation < 10 degrees) throughout flexion-extension; and a spiraling LoNE trajectory exists in the distal shank. The layout is accordingly organized as follows: core channels (A1, A3, K1, K3) are mounted perpendicular to the local LoNE to maximize signal-to-noise ratio; exploratory channels (A2, A4, K2, K4) are offset by 45 degrees at the same anatomical site to capture non-sagittal-plane motion via projection-component variation; a pair of channels is placed on the medial and lateral sides of the knee to extract inversion-eversion components through bilateral signal differencing; and A5 is mounted along the spiraling LoNE at the mid-shank, serving simultaneously as a pickup for knee-ankle coupling motion and an experimental checkpoint for the LoNE zero-strain property. The positions, orientations, and design rationale for all nine sensors are listed in Table 1, and Figure 1b annotates the anatomical location and LoNE-referenced attachment angle of each channel on anterior and posterior views of the lower limb.

To efficiently convert the strain information collected by the nine channels into a gait-class decision, a linear classifier provides the most direct mathematical formulation. The output voltages of N channels form an input vector x, and the decision function is

(2) $$y=w^{T}x+b=\Sigma_{i}w_{i}x_{i}+b$$

The core operation of Equation (2) is a weighted summation, which has a natural physical counterpart in a resistive network. If N branch currents are directed into a common node, with each branch current $I_{i}=G_{i}\cdot x_{i}$ being the branch transconductance, then, by Kirchhoff’s current law (KCL), the total node current is $I_{\mathrm{sum}}=\sum_{i}\left(G_{i}x_{i}\right)$, which is mathematically identical to Equation (2): the sensor output voltage acts as the input $x_{i}$, the branch conductance as the weight $w_{i}$, the nodal current superposition performs the summation, and the total current is the classification output y [22,23]. For a K-class task, it suffices to set K − 1 ordered thresholds on the output y, partitioning it into K intervals, each corresponding to a gait class. Consequently, the entire path from sensor response to classification decision is closed within the analog domain; no analog-to-digital conversion or digital computation is required, and the inference latency is limited only by the bandwidth of the analog components.

As shown in Figure 1c, the overall system comprises three layers: a skin-mounted sensor array, an analog-domain ISC signal chain, and a digital readout with Bluetooth transmission module.

The analog signal chain consists of four functional stages and a current-summing node. Stage 1 is the resistive-divider detection stage: each of the nine sensor channels (A1–A5, K1–K4) is connected in series with a fixed resistor $R_{i}$ to $V_{\mathrm{CC}}$; as the sensor resistance varies with skin deformation, the divider-node voltage changes accordingly. The static resistance of the liquid-metal sensors is approximately 33 Ω, and the reference resistors are chosen at the same order of magnitude to keep the divider operating in its sensitive range. Stage 2 is the instrumentation-amplifier stage, which differentially amplifies the millivolt-level divider changes to a range processable by subsequent stages. Stage 3 is the polarity-encoding stage: depending on the sign of the offline-trained weight, each channel is routed to either a non-inverting follower or an inverting follower [22,23], preserving the original polarity for positive-weight channels and inverting it for negative-weight channels. Stage 4 is the magnitude-encoding stage, employing digital potentiometers whose wiper positions are written via SPI by the STM32 using control words $D_{i}$(0–127), yielding $V_{\mathrm{wiper}}=V_{\mathrm{follow}}\times D_{i}/128$ and thereby continuously scaling each branch voltage to represent the absolute value of its weight. After polarity and magnitude encoding, the nine branch currents superpose at a common node in accordance with KCL, and a transimpedance amplifier (TIA) converts the total current into an output voltage $V_{\mathrm{out}}=-R_{f}\times I_{\mathrm{sum}}$. Three daily gaits—walking, running, and descending stairs—are adopted as classification targets; their kinematic differences in joint excursion and cadence render the nine-channel signals linearly separable in feature space, and the TIA output current falls into three distinct intervals: approximately 26 mA for stair descent, 35 mA for walking, and 42 mA for running. Class assignment requires only fixed-threshold comparison.

Weight acquisition and deployment proceed in two steps. In the offline stage, a 9-input, 1-output, bias-free linear layer is trained in PyTorch (version 2.7.1, CUDA 11.8) on a PC, and the converged weights are mapped to potentiometer control words. In the online stage, the STM32 writes these control words channel by channel into the digital potentiometers over SPI; once programming is complete, the PC is disconnected, and the system enters autonomous operation.

The digital readout layer is based on an STM32F103C8T6 microcontroller (STMicroelectronics, Geneva, Switzerland), whose on-chip 12-bit ADC samples the TIA output at 200 Hz; a 50 ms sliding-window majority vote in firmware suppresses single-sample misjudgments. Classification labels are transmitted via a JDY-31 Bluetooth module (Guangzhou HC Information Technology Co., Ltd., Guangzhou, China) at 5 bytes per frame (header, class label, raw ADC value, checksum), corresponding to a link bandwidth of approximately 8 kbps. The entire unit is powered by a single 3.7 V/500 mAh lithium cell; the measured operating current is approximately 68.5 mA, yielding a battery life of about 7 h.

## 3. Sensor Fabrication and Performance Characterization

The stretchable strain sensors are fabricated by screen-printing a liquid-metal–polymer composite conductive ink onto a thermoplastic polyurethane (TPU) film substrate. The sensing region is approximately 20 mm long and adopts a grid pattern designed to balance sensitivity and stretchability.

Because the nine channels of the ISC system share a single pair of thresholds for three-class discrimination, sufficiently low device-to-device scatter in baseline resistance, sensitivity, and linearity is essential; otherwise, output drift caused by the same gait action across different channels would blur inter-class boundaries, increasing the burden on weight training or even introducing uncompensable errors. The sensors fabricated in the same batch were therefore characterized item by item.

**Figure 2:**
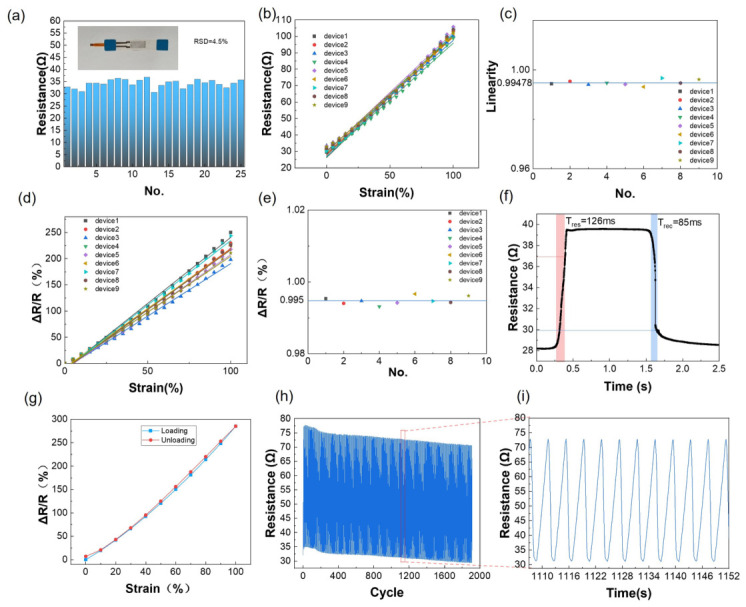
Performance characterization of the liquid-metal stretchable sensors. (**a**) Photograph of a single sensor and initial-resistance uniformity of 25 sensors (*RSD* = 4.5%). (**b**) Resistance-versus-strain curves for 9 sensors. (**c**) Linearity $R^{2}$ distribution. (**d**) Relative resistance change ΔR/R versus strain. (**e**) Uniformity of the fitting coefficient of determination ($R^{2}$) for nine flexible sensors under tensile strain. (**f**) Response time (126 ms) and recovery time (85 ms) under a step strain. (**g**) Hysteresis behavior: loading and unloading ΔR/R–strain curves over the 0–100% strain range, with a hysteresis index of ∼3.2%. (**h**) Cyclic stability over 2000 consecutive stretch–release cycles at 60% strain amplitude. (**i**) Magnified view of representative cycles from the red box in (**h**), showing the resistance response versus time.

Figure 2a shows a photograph of a single sensor: the grid-patterned liquid-metal conductive traces formed by screen printing are visible on the transparent TPU substrate, with blue conductive-tape pads leading out at both ends. Initial resistances of 25 sensors measured with a multimeter in the unloaded flat state are summarized in Figure 2a; the mean is approximately 33 Ω with a relative standard deviation (RSD) of 4.5%, indicating high baseline-resistance uniformity.

Tensile tests were carried out on the nine sensors designated for the array using a stretching platform. Figure 2b presents the resistance-versus-strain curves over the 0–100% strain range: the initial resistance is approximately 32 Ω, rising to 99–106 Ω at 100% strain, with all nine curves tracking closely without any individual device deviating from the group trend. In Figure 2d, the ordinate is converted to relative resistance change ΔR/R, which increases approximately linearly from 0 to 1.98–2.50. The gauge factor (GF), extracted as the slope of the ΔR/R versus strain linear fit over the 0–100% interval for each sensor, is plotted in Figure 2c; the mean GF of the nine devices is 2.27 with an RSD of 6.8%. Although a moderate inter-device GF scatter (*RSD* 6.8%) remains, it is automatically absorbed by the ISC offline weight-training step, which assigns each channel an independent weight and thereby compensates for inter-channel gain differences without manual calibration; the optimization can thus focus on discriminating gait features.

Figure 2e collects the coefficient of determination $R^{2}$ of the resistance–strain linear fits for the nine sensors; the mean reaches 0.99533 and the lowest individual value still exceeds 0.99, with an *RSD* of only 0.3%. The high linearity of the sensor response directly supports the mathematical premise of the linear classifier in the ISC: the closer the input-output relationship is to linear, the higher the precision with which the analog-domain weighted summation approximates the true decision function, and no additional nonlinearity-correction stage is needed in the circuit.

Loading–unloading tests were performed over the 0–100% strain range at a constant displacement rate of 1000 mm/min. As shown in Figure 2g, the ΔR/R–strain curves for the loading and unloading paths overlap closely; the maximum deviation between the two paths is below 2.8% of full scale, yielding a hysteresis index—defined as the ratio of the enclosed loop area to the loading-curve area—of approximately 3.2% (obtained by numerical integration). Within the 10–60% strain range relevant to gait monitoring, the deviation remains below 3%, confirming a highly repeatable response under realistic operating conditions. As shown in Figure 2f, step-strain transient measurements give a response time of 126 ms and a recovery time of 85 ms. Long-term cyclic stability was assessed over 2000 consecutive stretch–release cycles at a fixed strain amplitude of 60%, with resistance recorded continuously by a digital multimeter, as shown in Figure 2h. The peak resistance decreases gradually and monotonically from ∼77.9Ω (average of the first 100 cycles) to ∼71.8Ω (average of the last 100 cycles), corresponding to a total drift of about 7.6%. Critically, this drift is smooth and slowly varying, with no abrupt baseline jumps or electrical disconnections. Because the ISC front end employs online-rewritable digital potentiometers, such predictable slow drift can be fully accommodated by a periodic baseline recalibration routine that re-zeros each channel, without retraining the classification weights. All sensing data presented in this work represent raw multimeter output without any post-acquisition filtering or manual signal adjustment.

Taken together, the resistance *RSD* (4.5% over 25 devices), GF RSD (<5% over 9 devices), and linearity *RSD* (0.3% over 9 devices) all rank at favorable levels among reported flexible strain sensors, providing a reliable hardware-uniformity foundation for the nine-channel ISC system.

## 4. Experimental Methods

Five subjects (four male, one female; ages 21–25) participated in the experiments. For each subject, the nine sensors were attached to the ankle and knee regions of the right leg according to the layout in Table 1. Before testing, the skin was cleaned with medical-grade alcohol; after evaporation, the nine sensors were applied sequentially. Each subject then stood still for 30 s to acquire the baseline resistance of every channel.

Data acquisition was divided into two groups. The first group served to verify the LoNE-guided layout: subjects sat on an examination bed and performed ten single-degree-of-freedom and compound ankle movements (dorsiflexion, plantarflexion, inversion, eversion, dorsiflexion + inversion, dorsiflexion + eversion, plantarflexion + inversion, plantarflexion + eversion, clockwise circumduction, counterclockwise circumduction) together with three knee movements (leg raise, medial kick, lateral kick), each performed in three sets of three repetitions. The second group served for gait classification: subjects walked continuously along a corridor for 1 min, ran for 1 min, and then descended approximately 40 stair steps three times in succession. To assess repeatability and within-subject generalization, three subjects (S2–S4) completed the gait group over multiple independent sessions (3–5 sessions each, recorded on separate trials), whereas S1 and S5 each completed a single session. Both groups were sampled synchronously across all nine channels by the STM32 on-chip 12-bit ADC at 200 Hz.

Classifier training was first performed on a PC to verify the functionality of the in-sensor linear-classification circuit. With walking (T), running (Z), and stair descent (V) as the three class labels, a 9-input, 1-output, bias-free linear layer was optimized over 5000 iterations of stochastic gradient descent in PyTorch. The loss fell from an initial value of approximately 1100 to about 4.5 within the first 1000 iterations and then leveled off, confirming that the linear architecture converges stably and that the resulting weights can be realized in hardware. The converged weights were mapped to integer control words in the range 0–127 and written channel by channel into the digital potentiometers by the STM32 over SPI, completing the transfer from software weights to hardware gains. This procedure verifies the circuit-level classification function; the classification accuracy on real multi-subject gait signals, together with cross-validation, is reported separately in Section 5.

To evaluate classification performance on real multi-subject signals, the continuous nine-channel recordings (ankle A1–A5, knee K1–K4) were segmented into overlapping windows of 60 samples (≈1.5 s, 50% overlap). For each window, three features were extracted per channel—standard deviation, peak-to-peak amplitude, and zero-crossing rate (relative to the channel mean)—yielding a 27-dimensional feature vector that captures both the magnitude and the temporal rhythm of each gait pattern. Features were standardized to zero mean and unit variance using statistics computed from the training set, and a linear classifier (multinomial logistic regression) was trained to distinguish the three activities. Three complementary validation schemes were employed: (i) *within-subject cross-session validation*, training on a subject’s remaining sessions and testing on a held-out session of the same subject; (ii) *leave-one-subject-out (LOSO) cross-validation*, training on all subjects except one, and testing on the entirely unseen subject; and (iii) *calibrated cross-subject validation*, in which a small fraction of the target subject’s data is added to the training set to emulate per-user calibration.

## 5. Results and Discussion

**Figure 3:**
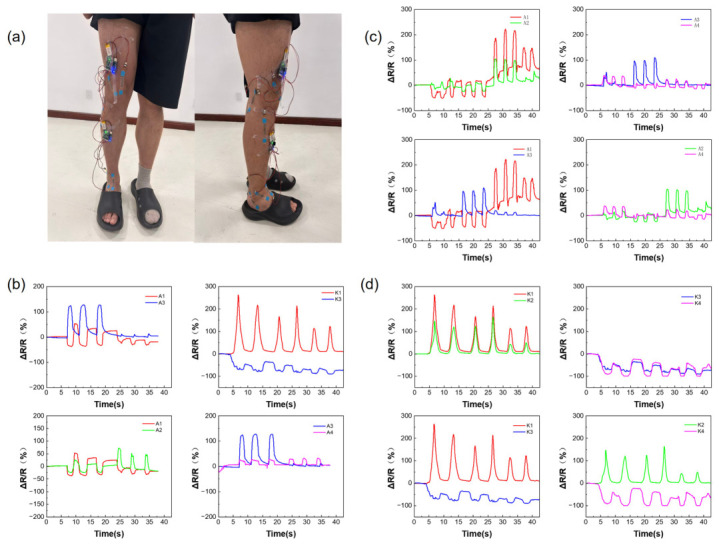
Experimental system and signal analysis. (**a**) Photograph of the sensor array connected to the ISC circuit. (**b**) Joint-motion test waveforms (including anti-phase verification, perpendicular vs. $45^{\circ }$ comparison, and A5 zero-strain verification). (**c**) Representative channel waveforms for ankle compound movements, including dorsiflexion combined with inversion/eversion and plantarflexion combined with inversion/eversion (three trials per motion). (**d**) Representative channel waveforms for knee movements, including leg raises, medial kicks, and lateral kicks (two trials per motion).

The soundness of the sensor layout requires experimental answers to three questions: do sensors on opposite sides of the LoNE exhibit the anti-phase response predicted by theory? Is there the expected amplitude difference between perpendicular-to-LoNE and 45-degree-offset sensors? Does A5, mounted along the LoNE, indeed approach zero response? The joint-motion waveforms in Figure 3 provide point-by-point verification.

A1 is attached to the anterolateral ankle and A3 to the Achilles-tendon surface, on opposite sides of the ankle LoNE [Figure 3b]. During dorsiflexion, the posterior skin is stretched, causing A3 to increase, while the anterior skin is compressed, causing A1 to decrease; the polarities reverse during plantarflexion. Pearson correlation of the two waveforms yields r=−0.98, closely matching the anti-phase prediction of LoNE theory. This property is exploited directly in the ISC circuit: by routing A1 and A3 to the non-inverting and inverting followers, respectively, the two branch currents add constructively at the KCL node, widening the inter-class output-current separation. At the same site, the perpendicular channel A1 and the 45-degree-offset channel A2 show a clear amplitude difference, with the peak-to-peak value of A1 being approximately 1.4 times that of A2, close to the reciprocal of $\cos45^{\circ }\approx 0.707$ projection attenuation.

The offset channels are not redundant, as the compound-movement experiments confirm. Figure 3c presents the responses to four compound ankle movements (dorsiflexion or plantarflexion combined with inversion or eversion). The perpendicular core channels dominate—A1 reaches approximately 265% during dorsiflexion + eversion—while the offset channels A2 and A4 produce smaller but distinctly shaped responses (A2/A1 ratios of 0.45–0.54). Crucially, A1 responds almost identically to the same primary motion regardless of the superimposed inversion or eversion component, whereas the offset channels resolve this difference, confirming that the 45-degree channels capture the non-sagittal-plane components missed by the perpendicular channels.

The knee channels show the same principle [Figure 3d] for three movements (leg raise, leg raise with inversion, and leg raise with eversion). The perpendicular channels K1 and K3 are strongly anti-phase—K1 produces positive peaks up to ∼263% while K3 shows negative-going responses down to ∼−90%—reflecting the opposite skin strains on the medial and lateral sides of the knee LoNE. The offset channel’s relative contribution rises during frontal-plane motion: the K2/K1 ratio increases to 0.80 during inversion, versus 0.53 for the plain leg raise and 0.44 for eversion. The three movements thus produce distinguishable four-channel signatures, and the medial–lateral difference provides a usable cue for the inversion–eversion direction.

**Figure 4:**
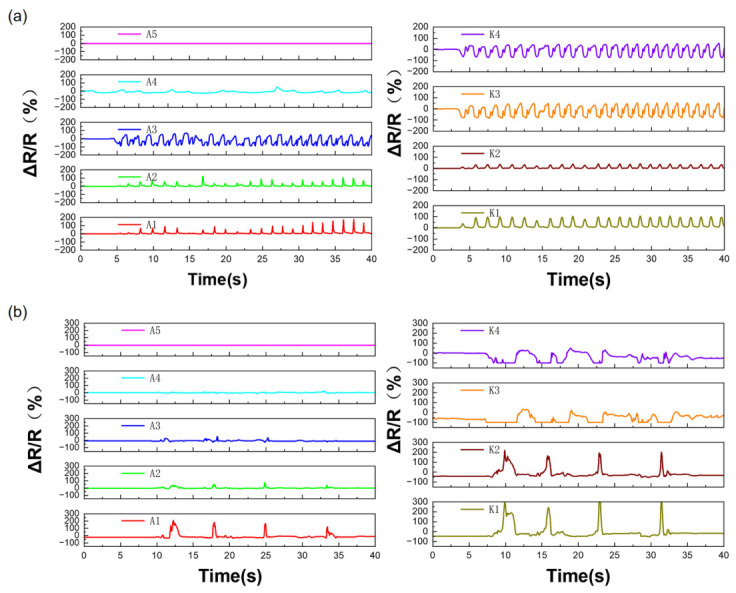
Multi-channel sensor responses during composite lower-limb movements. (**a**) Waveforms of all nine channels (ankle A1–A5 and knee K1–K4) during running. (**b**) Waveforms of all nine channels during a complex, non-periodic tennis swing. These results demonstrate that the system can effectively distinguish between periodic gait and aperiodic athletic motions based on their distinct spatiotemporal signatures.

Finally, A5, mounted along the spiraling LoNE at the mid-shank, verifies the zero-strain property. As shown in Figure 4a, during running, A1 and A3 exhibit pronounced periodic $\Delta R/R_{0}$ fluctuations on the order of 100%, whereas A5 varies by only a few percent, essentially remaining at baseline; the residual fluctuation arises from calf-muscle contraction and knee–ankle coupling. Figure 4 further contrasts this periodic gait with a complex, non-periodic movement (tennis swing): running [Figure 4a] yields regular, rhythmic peaks of consistent amplitude and interval, whereas the tennis swing [Figure 4b] produces irregular, large-amplitude bursts with no fixed period (K1 reaching ∼300%). This shows that the channel responses encode both the amplitude and the temporal structure of a movement, enabling the system to distinguish rhythmic gait from complex aperiodic actions.

**Figure 5:**
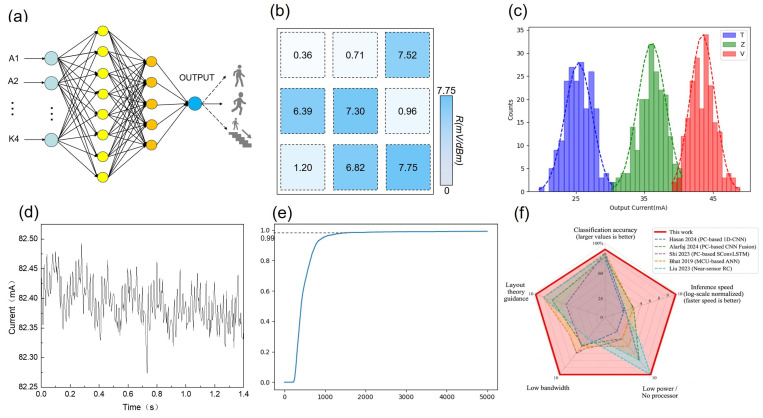
Classification performance and system comparison. (**a**) ISC network architecture (9 inputs, 1 output). Dashed lines indicate alternative routing options; only one of the three configurations is implemented in hardware. (**b**) Nine-channel weight heatmap. (**c**) TIA output-current histogram for the three gait classes. (**d**) Acquired circuit noise waveform. (**e**) Training accuracy curve. (**f**) Five-dimensional radar-chart comparison with reported schemes [24,25,26,27,28].

Figure 5c plots the TIA output current of the ISC circuit during the three gaits as a histogram. Stair-descent outputs cluster in the 22–30 mA range with a mean of approximately 25 mA; walking clusters in 30–38 mA with a mean of approximately 33 mA; running clusters in 36–45 mA with a mean of approximately 40 mA. The three distributions are tightly grouped, with only a small number of samples overlapping at adjacent class boundaries. Two thresholds partition the current axis into three intervals, yielding a classification accuracy of 99% at the circuit-functional-verification level, i.e., on the separable TIA-output distributions used to validate the analog classifier; the misclassified samples all lie in the transition band between walking and stair descent. The classification accuracy on real multi-subject gait signals, together with cross-validation, is evaluated separately below.

Figure 5b presents the converged channel weights as a heatmap. The largest values fall on the core channels perpendicular to the LoNE—the weights for the A1 region reach 7.52 and 7.75—while some $45^{\circ }$ exploratory channels carry weights as low as 0.36 and 0.71. This distribution was not manually prescribed but emerged automatically from gradient-based optimization, yet it aligns precisely with the expectation from LoNE theory: channels with the largest response amplitude receive the largest weights, whereas the along-LoNE channel with the weakest response is assigned a near-zero weight. In other words, the strain-direction prediction at the theoretical level, the signal-amplitude verification at the experimental level, and the weight assignment at the algorithmic level all point to the same set of channels, forming a self-consistent loop from physical model to hardware realization.

The training history is recorded in Figure 5e. The loss decreases from an initial value of approximately 1100 to about 4.5 within the first 1000 iterations and then plateaus; the accuracy at the circuit-functional-verification level rises in step, stabilizing at 99% after roughly 1000 iterations and remaining there through 5000 iterations. The rapid convergence and low final loss confirm that the linear architecture converges stably and that the resulting weights can be realized in hardware. This verifies the circuit-level classification function; performance on real multi-subject signals is reported below.

Figure 5d shows the TIA output current under a static input, characterizing the noise of the five-stage analog chain (TIA and digital potentiometers). The signal consists of a baseline current of approximately 82.3 mA with superimposed noise; the measured peak-to-peak noise is 0.41 mA, and the RMS noise is 0.069 mA, corresponding to a signal-to-noise ratio of about 61.5 dB (noise RMS about 0.08% of the baseline). This low noise level is far smaller than the inter-class separation of the classification output, and therefore does not affect gait-class discrimination.

To position the system performance across a broader set of metrics, Figure 5f compares the present system with five reported representative schemes along five dimensions: classification accuracy, inference speed, dependence on an external processor, transmission bandwidth, and availability of a theoretical layout basis. The comparison schemes span three typical architectures: PC-based deep learning—Hasan et al. [25] achieved 100% accuracy on four gait classes with a 1D-CNN, Alarfaj et al. [26] reached 94.8% on multiple activities with CNN fusion, and Shi et al. [27] attained approximately 96% with SConvLSTM; MCU-based edge inference—Bhat et al. [28] ran a lightweight ANN on a TI-CC2650 with 97.7% accuracy on six activities and 27.6 ms inference latency; near-sensor computing—Liu et al. [24] used reservoir dynamics of electrolyte-gated transistors for four-class gait recognition with an accuracy exceeding 93%.

In the accuracy dimension, the present system reaches 99% at the circuit functional level (87.0% under within-subject cross-session validation on real multi-subject data), falling within the same range as the compared schemes. In inference speed, the latency of PC-based schemes lies in the 50–200 ms range, the MCU-based scheme at 27.6 ms, and the near-sensor scheme at the millisecond level, whereas the analog-domain weighted summation of the present system completes in the microsecond range. Regarding processor dependence, all five comparison schemes require a digital processor (PC or MCU) for classification, while in the present system, classification is performed entirely in the analog circuit, with the STM32 responsible only for ADC readout and Bluetooth transmission. In transmission bandwidth, conventional schemes must relay multi-channel raw waveforms, whereas the present system transmits only class labels at 8 kbps. At the layout level, the sensor positions and orientations of all comparison schemes are empirically selected, whereas the present system uses LoNE theory as its quantitative layout basis. Table 2 summarizes these metrics in quantitative form.

Overall, the distinguishing feature of the present system lies in integrating theory-guided layout design, analog-domain front-end inference, and low-bandwidth wireless transmission within a single architecture, such that none of the five dimensions exhibits a pronounced weakness. This integration provides a reference technical path for the evolution of flexible strain sensors from single-point signal acquisition toward multi-channel real-time classification systems.

While the LM composite sensors fabricated here exhibit low hysteresis (∼3.2%) and a stable gauge factor within the 10–60% strain range relevant to lower-limb gait monitoring, it is important to contextualize these results against the known limitations of resistive strain sensors. Resistive sensors—regardless of conductive filler—are generally susceptible to baseline drift at higher strains, as reported for CNT/AgNP systems [7,8] and LM-particle composites [9,10]; our own devices show a gradual ∼7.6% baseline drift over 2000 cycles. In the present application, skin deformation during the three target gaits remains predominantly within 10–60% strain, a regime in which the sensors respond reliably and repeatably, which is one reason resistive LM sensors were selected for this proof-of-concept. For future extensions to higher-deformation scenarios, two mitigation strategies are worth noting. First, algorithmic compensation: the offline weight-training step of the ISC circuit already absorbs inter-channel gain differences, and a periodic recalibration routine could further correct slow drift by re-zeroing each channel’s baseline. Second, a transition to capacitive sensing: IDE-structured capacitive sensors based on liquid-metal channels have demonstrated significantly lower hysteresis and superior long-term stability than resistive counterparts [9], and are architecturally compatible with the KCL-based weighted-summation front end, since capacitance can be converted to a voltage via a capacitance-to-voltage interface stage before entering the existing signal chain. Exploring this substitution is a natural direction for future work aimed at broadening the applicable strain range of the ISC platform.

**Figure 6:**
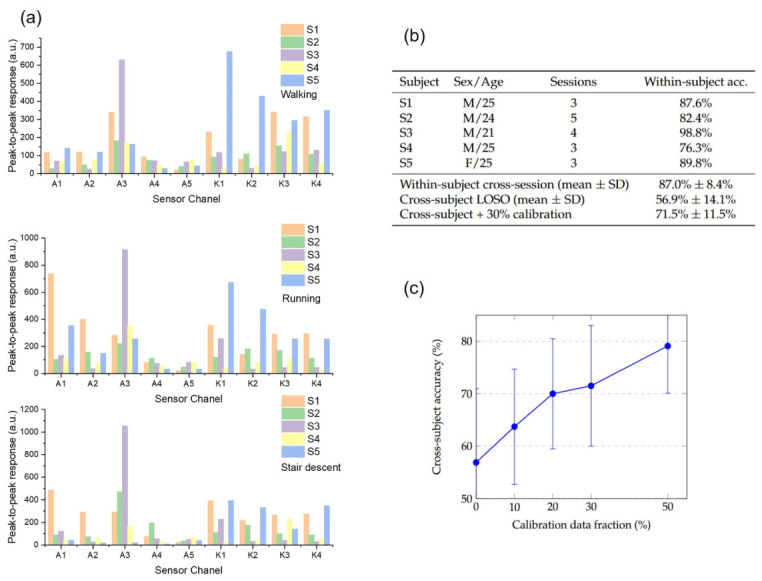
Analysis of sensor responses, subject characteristics, and calibration effects on cross-subject classification accuracy. (**a**) Comparison of sensor channel peak-to-peak responses across subjects during three movement modes. (**b**) Subject information and classification accuracy under three validation schemes. (**c**) Cross-subject accuracy versus the amount of per-user calibration data.

To rigorously assess inter-individual generalization and preclude overfitting beyond the circuit-level verification above, we evaluated classification performance on real multi-subject signals under three validation schemes (Figure 6b). Under intra-subject cross-session validation, the classifier trained on a subject’s earlier sessions and tested on a held-out session of the same subject achieved 87.0%±8.4% accuracy (S1: 87.6%, S2: 82.4%, S3: 98.8%, S4: 76.3%, S5: 89.8%). Importantly, because training and testing used data from different recording sessions of the same subject, this result demonstrates that the model captures stable, repeatable movement patterns rather than overfitting to a single recording.

Under leave-one-subject-out (LOSO) cross-validation, where the test subject was entirely unseen during training, the accuracy dropped to 56.9%±14.1%. This reduction reflects genuine inter-individual variability rather than a failure of the classifier. As shown in Figure 6a, the per-channel response patterns differ markedly across subjects: for the same activity, the dominant channels are not shared between individuals—some subjects are ankle-channel-dominant while others are knee-channel-dominant, and the relative channel amplitudes vary substantially from person to person. These differences arise from individual variations in limb geometry, sensor placement, and gait habits, so a weight vector trained on one set of subjects does not transfer directly to an unseen individual.

Such inter-subject variability is well documented for wearable strain-sensing systems and is commonly addressed by per-user calibration. Leveraging the online-rewritable digital potentiometers of the ISC front end, we added a small amount of the target subject’s data to the training set and re-trained only the linear weights, leaving the sensor layout and hardware unchanged. As shown in Figure 6c, the cross-subject accuracy rose monotonically with the amount of calibration data, increasing from 56.9% (no calibration) to 71.5%±11.5% with 30% calibration data and approaching 79% with 50%. This demonstrates that the reconfigurable architecture turns per-user calibration into a low-cost, practical operation, enabling reliable personalized recognition without any hardware modification, and provides direct experimental evidence for cross-subject weight adaptation.

## 6. Conclusions

This work introduces LoNE theory into the layout design of flexible strain sensors and constructs an analog-domain ISC classification front end, forming a complete technical chain from skin-mechanics-guided layout to analog-domain edge inference. The sensors, fabricated by screen-printing a liquid-metal–polymer composite ink on a TPU substrate, exhibit tensile linearity $R^{2}>0.99$ and sensitivity nonlinearity below 1%. The fabricated sensors exhibit a hysteresis index of ∼3.2% across the 0–100% strain range, a response/recovery time of 126/85 ms, and stable cyclic behavior over 2000 stretch–release cycles, with only gradual baseline drift of approximately 7.6%, confirming their suitability for continuous gait monitoring within the skin-deformation range of the target joint regions. The anti-phase experiments (r = −0.98), amplitude comparison between perpendicular and 45-degree channels, and the near-zero response of A5 all agree with the directional predictions of LoNE theory. The ISC circuit performs a nine-channel weighted summation in the analog domain in a single step; TIA output currents for the three gait classes center at approximately 25 mA, 33 mA, and 40 mA, yielding 99% two-threshold classification accuracy at the circuit-level functional-verification stage, with microsecond-level inference response and Bluetooth transmission of class labels only (8 kbps). On real multi-subject data, the system achieves 87.0%±8.4% accuracy under within-subject cross-session validation, and per-user weight calibration further raises cross-subject accuracy from 56.9% to 71.5%. Channel-ablation experiments confirm that each sensor tier makes a distinguishable contribution to classification accuracy. The three-gait classification task serves as a proof of concept, validating the feasibility of combining LoNE-guided layout with analog-domain ISC under real-world wearing conditions. Because LoNE theory applies to any joint region of the body where a quantifiable strain field exists, the same layout methodology can be extended to the shoulder, elbow, or spinal segments. The KCL-based linear-classification circuit architecture is independent of the specific sensor material; an increase in the number of target classes can be accommodated through multiple TIA outputs or hierarchical threshold strategies. The online-rewritable nature of the digital potentiometers also preserves a hardware interface for cross-subject weight adaptation. As the analog signal chain evolves toward ASIC integration and fully flexible packaging, this scheme holds promise as a low-power, lightweight wearable platform for multi-joint motion monitoring, gait-abnormality screening, and real-time motion feedback.

## Figures and Tables

**Table 1:** LoNE-guided nine-sensor multi-angle layout scheme.

ID	Anatomical Site	Orientation	LoNE Source	Tier	Target Signal
A1	Anterolateral ankle	Perp. to LoNE	Marreiros L6	Core	Ankle dorsi-/plantarflexion
A2	Anterolateral ankle	LoNE + 45°	Marreiros L6	Exploratory	Lateral rotational component
A3	Achilles tendon	Perp. to LoNE	Marreiros L4	Core	Plantar-/dorsiflexion (anti-phase)
A4	Achilles tendon	LoNE + 45°	Marreiros L4	Exploratory	Achilles rotational component
A5	Mid-shank lateral	Along spiral LoNE	Marreiros/Wessendorf	Coupling	Knee–ankle coupling/LoNE check
K1	Lateral knee	Perp. to LoNE	Wessendorf	Core	Knee flexion–extension
K2	Lateral knee	LoNE + 45°	Wessendorf	Exploratory	Lateral rotational component
K3	Medial knee	Perp. to LoNE	Wessendorf	Core	Knee flexion–extension
K4	Medial knee	LoNE + 45°	Wessendorf	Exploratory	Medial rotational component

**Table 2:** Multi-dimensional performance comparison with representative schemes.

Scheme	Sensor Type	Classification	Accuracy	Latency	Processor	Layout Theory
Hasan 2024 [25]	PLM hydrogel	1D-CNN (PC)	100%	∼50 ms	Yes (PC)	None
Alarfaj 2024 [26]	IMU	CNN fusion (PC)	94.8%	∼100 ms	Yes (PC)	None
Shi 2023 [27]	Multimodal IMU	SConvLSTM (PC)	∼96%	∼200 ms	Yes (PC)	None
Bhat 2019 [28]	Textile + accel.	ANN (MCU)	97.7%	27.6 ms	Yes (MCU)	None
Liu 2023 [24]	EGT pressure	Reservoir comp.	>93%	∼ms	Yes	None
This work	LM × 9	ISC analog	99%	∼μs	No	LoNE

## Data Availability

The original contributions presented in the study are included in the article. Further inquiries can be directed to the corresponding authors.

## References

[1] Lam M.H., Fong D.T.P., Yung P.S.H., Chan K.M. (2009). Knee stability assessment on anterior cruciate ligament injury: Clinical and biomechanical approaches. BMC Sports Sci. Med. Rehabil..

[2] Li S., Francisco G.E., Zhou P. (2018). Post-stroke hemiplegic gait: New perspective and insights. Front. Physiol..

[3] Rubenstein L.Z. (2006). Falls in older people: Epidemiology, risk factors and strategies for prevention. Age Ageing.

[4] Patel S., Park H., Bonato P., Chan L., Rodgers M. (2012). A review of wearable sensors and systems with application in rehabilitation. J. Neuroeng. Rehabil..

[5] Reaz M.B.I., Hussain M.S., Mohd-Yasin F. (2006). Techniques of EMG signal analysis: Detection, processing, classification and applications. Biol. Proced. Online.

[6] Amjadi M., Kyung K.U., Park I., Sitti M. (2016). Stretchable, skin-mountable, and wearable strain sensors and their potential applications: A review. Adv. Funct. Mater..

[7] Gu J., Kwon D., Ahn J., Cho H., Park J., Kim J., Kim S., Park I. (2019). Wearable strain sensors using light transmittance change of carbon nanotube-embedded elastomers with microcracks. ACS Appl. Mater. Interfaces.

[8] Min S.H., Lee G.Y., Ahn S.H. (2019). Direct printing of highly sensitive, stretchable, and durable strain sensor based on silver nanoparticles/multi-walled carbon nanotubes composites. Compos. Part B Eng..

[9] Li X., Rytkin E., Zhao Q., Bhat P., Pfenniger A., Yin L., Huang X., Yang L., Yang B., Burrell A. (2025). High-resolution liquid metal–based stretchable electronics enabled by colloidal self-assembly and microtransfer printing. Sci. Adv..

[10] Liu S., Shah D.S., Kramer-Bottiglio R. (2021). Highly stretchable multilayer electronic circuits using biphasic gallium-indium. Nat. Mater..

[11] Iberall A.S. (1964). The Use of Lines of Nonextension to Improve Mobility in Full-Pressure Suits.

[12] Bethke K. (2005). The Second Skin Approach: Skin Strain Field Analysis and Mechanical Counter Pressure Prototyping for Advanced Spacesuit Design. Ph.D. Thesis.

[13] Wessendorf A.M., Newman D.J. (2012). Dynamic understanding of human-skin movement and strain-field analysis. IEEE Trans. Biomed. Eng..

[14] Marreiros S.S.P. (2010). Skin Strain Field Analysis of the Human Ankle Joint. Master’s Thesis.

[15] Choi J., Hong K. (2015). 3D skin length deformation of lower body during knee joint flexion for the practical application of functional sportswear. Appl. Ergon..

[16] Obropta E.W., Newman D.J. A comparison of human skin strain fields of the elbow joint for mechanical counter pressure space suit development. Proceedings of the IEEE Aerospace Conference.

[17] Wang L., Sun Y., Li Q., Zhang D., Zhao X. (2020). Latest research trends in gait analysis using wearable sensors and machine learning: A systematic review. IEEE Access.

[18] Ordóñez F.J., Roggen D. (2016). Deep convolutional and LSTM recurrent neural networks for multimodal wearable activity recognition. Sensors.

[19] Wan T., Wan B., Ma S., Zhou Y., Li Q., Chai Y. (2023). In-sensor computing: Materials, devices, and integration technologies. Adv. Mater..

[20] Prezioso M., Merrikh-Bayat F., Hoskins B.D., Adam G.C., Likharev K.K., Strukov D.B. (2015). Training and operation of an integrated neuromorphic network based on metal-oxide memristors. Nature.

[21] Mennel L., Symonowicz J., Wachter S., Polyushkin D.K., Molina M., Mueller T. (2020). Ultrafast machine vision with 2D material neural network image sensors. Nature.

[22] Gao W., Tai J., Xiang Z., Chen X., Li H. (2025). Temperature field ultrafast detection and identification quantum sensor based on diamond array. Microsyst. Nanoeng..

[23] Gao W., Tai J., Wang Z., Chen X., Li H. (2025). Diamond-neural-network magnetic sensors for ultrafast circuit fault detection and identification. Photonics Res..

[24] Liu X., Sun C., Guo Z., Zhang H., Wang L. (2023). Near-sensor reservoir computing for gait recognition via a multi-gate electrolyte-gated transistor. Adv. Sci..

[25] Hasan S., D’auria B.G., Mahmud M.A.P., Chowdhury M.E.H. (2024). AI-aided gait analysis with a wearable device featuring a hydrogel sensor. Sensors.

[26] Alarfaj M., Al Madini A., Alsafran A., Alruwaili M., Almutairi N. (2024). Wearable sensors based on artificial intelligence models for human activity recognition. Front. Artif. Intell..

[27] Shi L.F., Liu Z.Y., Zhou K.J., Wang Y. (2023). Novel deep learning network for gait recognition using multimodal inertial sensors. Sensors.

[28] Bhat G., Deb R., Chaurasia V.V., Ogras U.Y. Online human activity recognition using low-power wearable devices. Proceedings of the IEEE/ACM International Conference on Computer-Aided Design (ICCAD).

